# Challenging the negative learning bias hypothesis of depression: reversal learning in a naturalistic psychiatric sample

**DOI:** 10.1017/S0033291720001956

**Published:** 2022-01

**Authors:** Sophie C. A. Brolsma, Janna N. Vrijsen, Eliana Vassena, Mojtaba Rostami Kandroodi, M. Annemiek Bergman, Philip F. van Eijndhoven, Rose M. Collard, Hanneke E. M. den Ouden, Aart H. Schene, Roshan Cools

**Affiliations:** 1 Donders Institute for Brain, Cognition and Behaviour, Radboud University Medical Center, Nijmegen, the Netherlands; 2 Department of Psychiatry, Radboud University Medical Center, Nijmegen, the Netherlands; 3 Depression Expertise Centre, Pro Persona Mental Health Care, Nijmegen, the Netherlands; 4 School of Electrical and Computer Engineering, University of Tehran, Tehran, Iran

**Keywords:** comorbidity, computational model, depression, negative learning bias, reversal learning

## Abstract

**Background:**

Classic theories posit that depression is driven by a negative learning bias. Most studies supporting this proposition used small and selected samples, excluding patients with comorbidities. However, comorbidity between psychiatric disorders occurs in up to 70% of the population. Therefore, the generalizability of the negative bias hypothesis to a naturalistic psychiatric sample as well as the specificity of the bias to depression, remain unclear. In the present study, we tested the negative learning bias hypothesis in a large naturalistic sample of psychiatric patients, including depression, anxiety, addiction, attention-deficit/hyperactivity disorder, and/or autism. First, we assessed whether the negative bias hypothesis of depression generalized to a heterogeneous (and hence more naturalistic) depression sample compared with controls. Second, we assessed whether negative bias extends to other psychiatric disorders. Third, we adopted a dimensional approach, by using symptom severity as a way to assess associations across the sample.

**Methods:**

We administered a probabilistic reversal learning task to 217 patients and 81 healthy controls. According to the negative bias hypothesis, participants with depression should exhibit enhanced learning and flexibility based on punishment *v.* reward. We combined analyses of traditional measures with more sensitive computational modeling.

**Results:**

In contrast to previous findings, this sample of depressed patients with psychiatric comorbidities did not show a negative learning bias.

**Conclusions:**

These results speak against the generalizability of the negative learning bias hypothesis to depressed patients with comorbidities. This study highlights the importance of investigating unselected samples of psychiatric patients, which represent the vast majority of the psychiatric population.

## Introduction

Major depressive disorder (MDD) is a highly debilitating psychiatric condition, with an estimated yearly prevalence of 4.4% worldwide (WHO, 2017). Prior studies that have attempted to clarify the neurobiological and cognitive mechanisms underlying depression, mainly focused on selected patient samples, that either did not have comorbid psychiatric disorders or these disorders were not described (Admon et al., 2017; Elliott, Sahakian, Herrod, Robbins, and Paykel, 1997; Harlé, Guo, Zhang, Paulus, and Yu, 2017; Liu et al., 2017; Robinson, Cools, Carlisi, Sahakian, and Drevets, 2012; Rothkirch et al., 2017; Taylor Tavares et al., 2008). The current paper aims to extend these results by investigating a heterogeneous sample of depressed patients, with a high and well-defined level of comorbidities.

MDD has long been characterized by an imbalance between decreased reward and increased punishment sensitivity (Admon & Pizzagalli, 2015; Eshel & Roiser, 2010). One of the two key symptoms of MDD is anhedonia, which, according to the Diagnostic and Statistical Manual of Mental Disorders 5th edition (DSM-5; American Psychiatric Association, 2013) refers to a diminished interest or pleasure (in almost all activities). Translated towards reward mechanisms, this can be understood as a reduced capacity to anticipate and experience pleasure from reward. A considerable amount of research has focused on reward processing in depression, and in general, these studies find reward learning deficits; a blunted response towards rewarding information and decreased reward sensitivity (Admon & Pizzagalli, 2015; Eshel & Roiser, 2010; Robinson et al., 2012; Safra, Chevallier, & Palminteri, 2019; Timmer, Sescousse, van der Schaaf, Esselink, & Cools, 2017). For example, when asked to respond to certain pictures, never-depressed individuals respond faster to pictures that have been rewarded more often during previous trials. MDD patients do not show this biased learning, implying they do not learn from reward as well as never-depressed individuals (Pizzagalli, Iosifescu, Hallett, Ratner, & Fava, 2008b). This learning deficit has also been shown in individuals who were remitted from depression, indicating that a previous depressive episode may have an enduring effect on reward learning (Pechtel, Dutra, Goetz, & Pizzagalli, 2013; Whitton et al., 2016).

Another key symptom of MDD is increased sensitivity to negative information, a characteristic also termed negative bias (Eshel & Roiser, 2010; Robinson et al., 2012). Several studies have investigated the negative bias hypothesis by using a probabilistic reversal learning (PRL) paradigm, a computer task which measures sensitivity to punishment and reward feedback. These studies observed enhanced sensitivity to punishment in MDD: depressed individuals exhibited greater tendency to reverse responding upon punishment relative to reward (Murphy, Michael, Robbins, & Sahakian, 2003; Taylor Tavares et al., 2008). This negative learning bias is consistent with the larger body of literature on negative information processing biases in MDD in the cognitive domains of attention, interpretation, and memory (Everaert, Podina, & Koster, 2017; Gotlib & Joormann, 2010; LeMoult & Gotlib, 2019; Mathews & MacLeod, 2005; Vrijsen et al., 2017). Negative bias research has generally focused on the processing of emotional words and pictures, e.g. self-descriptive words, emotional expressions. Thus, individuals show biased learning from positive and negative feedback but also differences in preferential processing of positive and negative emotional information. Furthermore, it is assumed that explicit feedback (such as the word ‘correct’ or ‘incorrect’) may still be interpreted with emotional quality, and can influence for example motivation (Roiser & Sahakian, 2013). However, most of these studies used selected samples (with regard to comorbidity, severity, age and/or medication), which limits the generalizability of the findings. Furthermore, they mostly used course, aggregate measures of behavior (i.e. participant learning scores) (Murphy et al., 2003; Robinson et al., 2012; Taylor Tavares et al., 2008).

The use of computational models might be a more sensitive approach to detect latent biases in trial-by-trial behavior (Robinson & Chase, 2017). Accordingly, in the present study, we recruited a large naturalistic sample of psychiatric patients, characterized by well-diagnosed comorbidity of a number of common psychiatric disorders, i.e. MDD, anxiety disorder, addictive disorder, attention-deficit/hyperactivity disorder (ADHD), and autism spectrum disorder (ASD). Investigating mechanisms of depression in a more ecologically valid group of patients (Goldberg & Fawcett, 2012; Kessler et al., 2003; Lamers et al., 2011; Rommelse, Geurts, Franke, Buitelaar, & Hartman, 2011) also allows us to assess whether the deficit is specific to depression, or reflects nonspecific psychiatric vulnerability.

We combined analyses of classic aggregate behavioral measures of punishment and reward sensitivity to enable comparison with prior work (Murphy et al., 2003; Taylor Tavares et al., 2008) with computational reinforcement learning modeling (den Ouden et al., 2013). This modeling allowed us to compute parameters reflecting positive and negative learning rate as well as decision variability. Learning rate indexes the degree to which people update their expectations about reward or punishment based on having received unexpected rewards and punishments in the past, in short, their speed of learning from experience. Decision variability indexes the degree to which choices are in line with their expectations, with high variability corresponding to high choice randomness putatively reflecting poor ability to translate value into action. Critically, recent studies with selected MDD samples using a similar approach have observed enhanced decision variability rather than changes in reward or punishment learning rate (Harlé et al., 2017; Huys, Pizzagalli, Bogdan, & Dayan, 2013; Kunisato et al., 2012) and have associated this with increased ratings of anhedonia.

We compared aggregate behavioral measures and model-based parameters of reward and punishment sensitivity adopting two strategies. First, we used a classic group comparison strategy: we contrasted patients with MDD, patients without MDD and healthy controls (HC). Second, we adopted a dimensional approach in line with the recommendations of Research Domain Criteria (RDoC) guidelines (Insel et al., 2010; National Institute of Mental Health, 2008), which aims to stimulate patient group stratification based on core brain-behavior dimensions rather than discrete diagnostic categories. We assessed the relationship between the outcome variables and symptom severity across the whole group (controls and patients), as measured with questionnaires.

Next, we investigated the specificity of the effects to MDD, by performing an additional set of analyses. First, we examined whether parallel effects were observed when stratifying the group by the other diagnoses present in our sample. Additionally, we assessed whether any of the effects could be accounted for by (i) medication use, or (ii) general psychiatric disease severity, in terms of the total number of other diagnoses. Finally, we examined whether negative learning bias was present in a patient subsample that was matched to previous studies based on age, comorbidity and medication use. This enabled more direct comparison with previous results.

## Methods

### General procedure

The present study is part of a cohort-study run by the Department of Psychiatry of the Radboud university medical center (Radboudumc), Nijmegen, The Netherlands. The MIND-Set study (Measuring Integrated Novel Dimensions in Neurodevelopmental and Stress-related Mental Disorders) is an ongoing observational cross-sectional study that assesses clinical, biological, behavioral, and neuroimaging data (online Supplementary Table S1). Data collection include a set of neuropsychological measures among which a PRL task, which was used to answer the current research questions.

All adult outpatients (age range 18–78, mean age of 40) with a diagnosis of a current depressive disorder (MDD or dysthymia), anxiety disorder, addictive disorder, ADHD and/or ASD were eligible for participation in the MIND-Set study. The present study was conducted during the DSM-IV/DSM-5 transition period. Diagnoses of MDD, dysthymia, anxiety disorder, addictive disorder, and ADHD were therefore established by DSM-IV criteria, and ASD by DSM-5 criteria. Depressive disorders, anxiety disorders and psychotic disorders were assessed with the Structured Clinical Interview for DSM-IV AXIS I Disorder (SCID-I; First, Spitzer, Gibbon, and Williams, 1996); ADHD with the Diagnostic Interview for Adult ADHD, second edition (DIVA 2.0; Kooij and Francken, 2010); ASD with the Dutch Diagnostic Interview for Adult Autism Spectrum Disorders (NIDA; Vuijk, 2016); and addictive disorders with the Measurements in the Addictions for Triage and Evaluation and criminality (MATE-crimi; Schippers, Broekman, and Buchholz, 2011).

Participants were excluded if they had a current psychotic disorder according to the SCID section B, an IQ estimation <70, a sensorimotor disability intervening with participation, were mentally incompetent to sign informed consent or had insufficient knowledge of the Dutch language. The study has been approved by the local ethics committee (Commissie Mensgebonden Onderzoek Arnhem-Nijmegen, NL 55618.091.015). Written informed consent for participating in this study was obtained after the diagnostic procedure (online Supplementary Methods).

### Participants

**Fig. 1.**
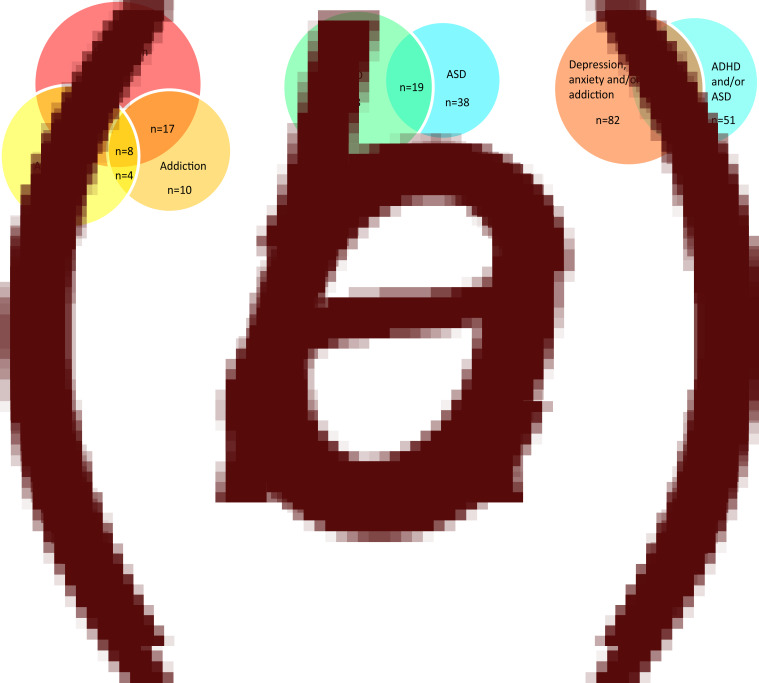
Overlap of disorders in the patient sample. (a) Overlap between patients with depressive disorders, anxiety disorders and addictive disorders. (b) Overlap between patients with ADHD and ASD. (c) Overlap between depressive, anxiety and addictive disorders on the one hand, with ADHD and ASD on the other hand. This overview did not include 20 patients with only remitted MDD or without a diagnosis at the time of inclusion.

For the current project, we included data from patients who were enrolled between June 2016 and December 2017. In this timeframe, 311 patients were included in the study, of whom 217 participated in the neuropsychological assessment (see online Supplementary Table S2 for an overview of the sample size per diagnostic category). This sample was divided into three groups: (i) the No MDD group (*n* = 61, patients with disorders other than current or remitted depression), (ii) the Remitted MDD group (*n* = 55, patients which had at least one previous depressive episode but did not meet the criteria for a depressive episode at the time of inclusion), (iii) the Current MDD group [*n* = 101, patients with a current depressive disorder (with or without past depressive episodes)]. This division took into account a possible vulnerability from previous depressive episodes in the patients who had remitted from depression. Comorbidity with other disorders (anxiety and/or addiction and/or ADHD and/or ASD) was possible in all three groups (Fig. 1).

In addition, healthy controls were included from October 2016 until June 2019, during which the data of 101 participants were collected. In the current study, we were able to match 81 healthy participants with no current or lifetime psychiatric diagnosis to the patient sample based on age, gender and education level. Healthy controls underwent the same testing procedure as the patients (see online Supplementary Methods).

### Probabilistic reversal learning task

**Fig. 2.**
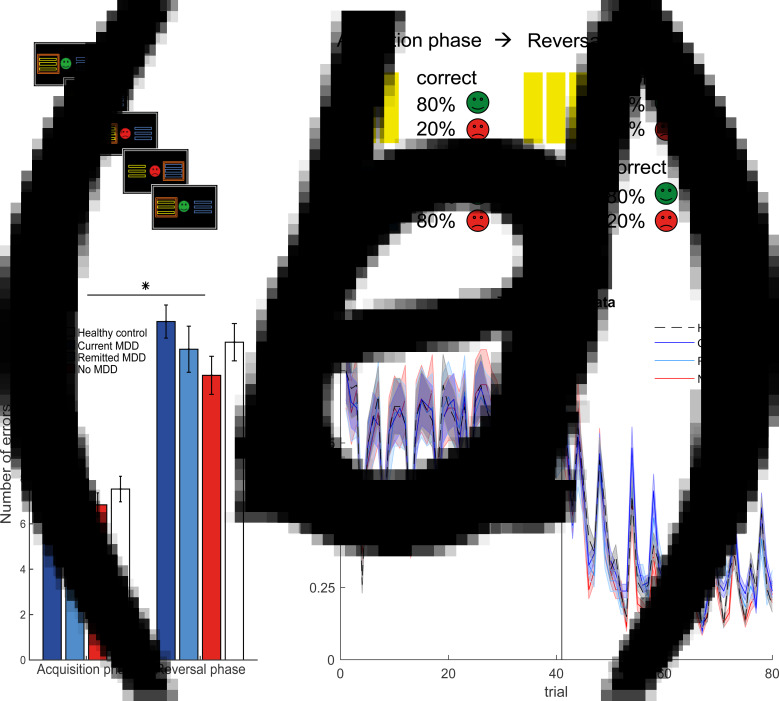
Task description and error rates. (a) Probabilistic reversal learning task. At the beginning of each trial, a yellow and a blue square were presented in two of four possible locations (top, bottom, left or right). Participants had to choose one of the squares, by pressing the corresponding arrow key on the keyboard (left, right, up, down). Squares were shown until a response was given. Subsequently, the feedback was given, which could be a reward (a green smiley accompanied by a high sound) or a punishment (a red sad smiley accompanied by low sound), which was shown for 1500 ms. The next trial started after 1000 ms. (b) During the acquisition phase, the square that was selected first (here yellow) would be rewarded 80% of the trials. During the reversal phase, the previous punished square would now be rewarded 80% of the trials. (c) The mean number of errors per group during the acquisition and reversal phase of the task. (d) The calculated trial-by-trial probability of choosing yellow (the square that was chosen on the first trial) per group. Shade represents the SEM. At trial 41 the contingencies were reversed. See for a similar figure of the simulated data online Supplementary Figure S1.

Subjects performed a probabilistic reversal learning task, a well-established paradigm investigating learning and behavioral adaptation based on reward and punishment (Cools, Barker, Sahakian, & Robbins, 2001; den Ouden et al., 2013; Swainson et al., 2000) (Fig. 2a). During the task, participants were presented with two squares. They needed to choose one, after which they received feedback; either a reward or a punishment. Subjects were instructed to choose the square that was rewarded most often. They needed to learn this by trial-and-error (see online Supplementary Material for subject instruction). The feedback was probabilistic: the square selected by the participant on the first trial was considered the correct square and rewarded on 80% of the trials. In the remaining 20% of the cases, selecting the same square was punished (despite the response being correct). This punishment feedback was therefore misleading, and should be ignored (participants should not switch responses). This was not explicitly stated. After the first 40 trials of the task, the ‘acquisition phase’, a ‘reversal phase’ started (also 40 trials). The probabilities switched in this phase (unbeknownst to the participants) so that the previously rewarded square was now punished on 80% of the trials (and vice-versa, the previously punished square was now rewarded on 80% of the trials). Before starting the experiment, we informed participants that the correct response could change, but they were not aware of how often or when this would occur (Fig. 2b).

### Additional measures

#### Socio-demographic information

Information on age, gender and level of education was obtained. Education was divided into four levels; (almost) no education (elementary education or education not finished), low (lower vocational and general secondary education), middle (intermediate vocational and higher secondary education) and high (higher vocational education and university) (Ikram et al., 2015).

#### Psychiatric symptom severity

Depressive symptoms were measured using the 30-item Inventory of Depressive Symptomatology self-report version (IDS-SR, Rush, Gullion, Basco, Jarrett, and Trivedi, 1996), anxiety with the Anxiety Sensitivity Index (ASI, Rodriguez, Bruce, Pagano, Spencer, and Keller, 2004), ADHD with the Conners’ Adult ADHD Rating Scale (CAARS, Conners, Erhardt, and Sparrow, 1999), and ASD with the Autism-spectrum Quotient (AQ-50; Baron-Cohen, Wheelwright, Skinner, Martin, and Clubley, 2001; Hoekstra, Bartels, Cath, and Boomsma, 2008). There was no separate questionnaire for the severity of addictive symptoms available.

#### Background neuropsychology

Verbal IQ was determined with the National Adult Reading Test score (NART score, Dutch version, Schmand, Lindeboom, and Van Harskamp, 1992). To assess the functional specificity of any effects, we also measured the total number of errors from the spatial working memory task (SWM errors) from the Cambridge Neuropsychological Test Automated Battery (CANTAB^®^; Cambridge Cognition 2019) to measure working memory capacity as an additional measure of cognitive ability.

#### Medication

Medication use was assessed during the clinical diagnostic procedure, and again during the neuropsychological assessment to monitor changes in medication use (online Supplementary Table S1). Of the 218 patients, 55 used a selective serotonin reuptake inhibitor (SSRI), 36 used benzodiazepines, 22 used antipsychotics, 18 used dopaminergic medication (e.g. methylphenidate), 12 used opioids, 9 used tricyclic antidepressants, and 4 used lithium at the time of the neuropsychological assessment. There were 65 participants who did not use any medication.

### Aggregate behavioral outcome measures

Based on previous work, we computed the following aggregate behavioral measures of reward and punishment learning and reversal:

- Error rate: the total number of errors during the acquisition and reversal phase (*z*-transformed);
- Probabilistic switch rate, defined as the number of errors after misleading feedback divided by the total number of misleading feedback trials (Murphy et al., 2003; Taylor Tavares et al., 2008);
- Win-stay and lose-shift rates, computed as the proportion of trials after a reward (*v.* punishment) on which the same square was chosen again (*v.* not chosen again), irrespective of whether this was the correct square or not (den Ouden et al., 2013).
- Perseveration errors, computed as any sequence of two or more errors in the reversal phase (*z*-transformed) (den Ouden et al., 2013). This outcome measure was taken as an index of behavior after the reversal.
- Number of participants that reached a learning criterion of eight consecutive correct responses during acquisition. Although it is an arbitrary measure, it has been used before (den Ouden et al., 2013; Swainson et al., 2000). It requires the participant to ignore at least two instances of misleading feedback.

### Computational modeling

In addition, we employed an established reinforcement learning model with dual learning rates, from here referred to as the reward-punishment (RP) model (Frank, Moustafa, Haughey, Curran, & Hutchison, 2007; Rescorla & Wagner, 1972). This approach estimates three key parameters: punishment learning rate, reward learning rate, and decision variability. Punishment (*v.* reward) learning rate reflects the degree to which participants update the value of an action depending on previous experience with unexpected punishment (*v.* reward). A high learning rate indexes greater weight on an unexpected outcome, thus faster updating of action value. Decision variability reflects the stochasticity of choices given this expected value and indexed by the softmax beta parameter: A high beta means lower decision variability (choosing the best option more consistently); a lower beta means that decisions are more random (see online Supplementary Materials for a detailed description).

### Statistical analyses

#### Demographic information

Gender, age, verbal IQ, education level, spatial working memory task (SWM) errors, and depressive symptom (IDS-SR total score) were submitted to an analysis of variance (ANOVA) to compare the four groups (No MDD, Remitted MDD, Current MDD, HC). A chi-square test was used to compare the total number of diagnoses between groups, as an index of general psychiatric severity (a maximum of six concurrent disorders: current MDD, remitted MDD, anxiety disorder, addictive disorder, ADHD, and ASD).

#### Behavioral outcome measures

Acquisition and reversal errors were submitted to repeated-measures ANOVA with the task phase as a within-subjects factor and group as a between-subjects factor. The proportion of subjects passing the learning criterion within each group was analyzed with a chi-square test. Win-stay and lose shift rate were submitted to repeated-measures ANOVA with error type as a within-subjects factor and group as a between-subjects factor. Model-based reward and punishment learning rate were also submitted to repeated-measures ANOVA with the learning rate as a within-subjects factor and group as a between-subjects factor. Finally, probabilistic switch rate, perseverative errors, and model-based decision variability were all submitted to separate ANOVAs with the group as a between-subjects factor.

#### Dimensional analyses

Spearman's partial correlations were computed across the whole sample, exploring the relationship between the different psychiatric symptom ratings (IDS-SR, ASI, CAARS, AQ-50) and the behavioral (probabilistic switch rate, win-stay, lose-shift) and computational (reward learning rate, punishment learning rate, decision variability) outcome measures.

We included age, gender and working memory capacity (SWM total errors) as covariates of no interest in all analyses. Significant effects (*p* value <0.05) were further investigated with follow-up *t* tests. Whenever there was unequal variance (measured with Levene's Test) between the groups, we present the results from the *t* tests that used the Welch-Satterthwaite correction, as implemented in SPSS. We used Bayesian ANOVAs to quantify the evidence in support of the null (no difference between groups) or alternative (a difference between patients and HC) hypotheses for the main behavioral and computational outcome measures (JASP Team, 2019).

#### Specificity analyses

We analyzed the outcome measures as a function of the other diagnoses present in our sample and compared them with the HC group. Behavioral and computational dependent variables were assessed with a multivariate ANOVA using either anxiety disorder (present/absent/HC), addictive disorder (present/absent/HC), ADHD (present/absent/HC) or ASD (present/absent/HC) as between-subjects factor. To correct for multiple comparisons we divided the *p* value by the number of tests, i.e. the number of outcome measures [4; error type (2 levels: Win-stay and lose-shift), probabilistic switch rate, learning rate (2 levels: Reward and punishment), decision variability] that were tested times the number of diagnoses (4; anxiety disorder, addictive disorder, ADHD, ASD), which was 0.05/16 equals a *p* value of 0.003.

Next, we explored whether any of the effects of diagnosis we observe can be accounted for by general psychiatric severity (indexed by the total number of diagnoses), or by type of medication used. We were specifically interested in the effects of the commonly used SSRIs, given our previous results from a genetic study on PRL (den Ouden et al., 2013). This study revealed an effect of common single nucleotide polymorphism in the gene encoding the serotonin transporter (SERT: 5HTTLPR plus rs25531) in the healthy subject population on the lose-shift rate. Therefore, we compared our outcome measures in patients who used an SSRI (*n* = 54), with patients who used other medication (*n* = 98), and with patients who did not use any medication (*n* = 65).

Furthermore, we specifically utilized the probabilistic switch rate (the number of errors after misleading feedback divided by the total number of misleading feedback trials) to enable comparison with two previous studies that examined PRL in depression. First, Murphy et al. (2003) examined 27 medicated MDD patients (age 26–59, antidepressant or mood-stabilizing medication) without comorbidities. Second, Taylor Tavares et al. (2008) examined 13 unmedicated MDD patients (18–55) also without comorbidity. These samples differ in sample size, medication use and comorbidity compared with the current sample. We therefore also analyzed probabilistic switch rate within a subsample of the HC and a subsample of the patients with comparable characteristics to the previous studies. Specifically, we analyzed the data of participants between 18 and 59 years old, and patients who only had a current MDD (recurrent or first episode) without any comorbidity. This yielded a sample of 24 patients, who were then divided based on whether they used an SSRI (*n* = 10) or not (*n* = 14). These patients were then compared with a subset of the HC in the same age range (*n* = 63). Although we could only compare probabilistic switch rate with the previous studies, we analyzed all outcome measures with Group (HC, Clean MDD no SSRI, Clean MDD SSRI) as between-subjects factor.

For completeness, we also report statistics for all direct comparisons, not corrected for multiple tests. Specifically, we compared all negative learning bias measures (i.e. probabilistic switch rate, lose-shift rate and punishment learning rate) between the following groups: Current MDD *v.* HC, Current MDD *v.* No MDD, and Current MDD *v.* Remitted MDD. See the online Supplementary Material for a detailed report.

Finally, to assess whether the findings were driven by individuals who may have used a different strategy to complete the task, or who did not completely understand the task, we restricted our analyses to participants who passed a learning criterion of eight consecutive correct responses in the acquisition phase. We examined the effect of Group and depressive symptoms on the different outcome measures, but only in participants who passed this criterion (online Supplemental Material).

## Results

### Probabilistic reversal learning is unimpaired in current and remitted MDD

#### Behavioral outcome measures

Error rate was higher during the reversal than the acquisition phase [main effect of Phase on number of errors: *F*(1,285) = 14.88, *p* < 0.001, *η*_p_^2^ = 0.05], but did not differ between the four groups [no main effect of Group: *F*(3,285) = 1.66, *p* = 0.177, *η*_p_^2^ = 0.02], and also not as a function of phase [interaction between Group and Phase: *F*(3,285) = 0.07, *p* = 0.975, *η*_p_^2^ = 0.001] (Figs 2c and 2d).

**Fig. 3.**
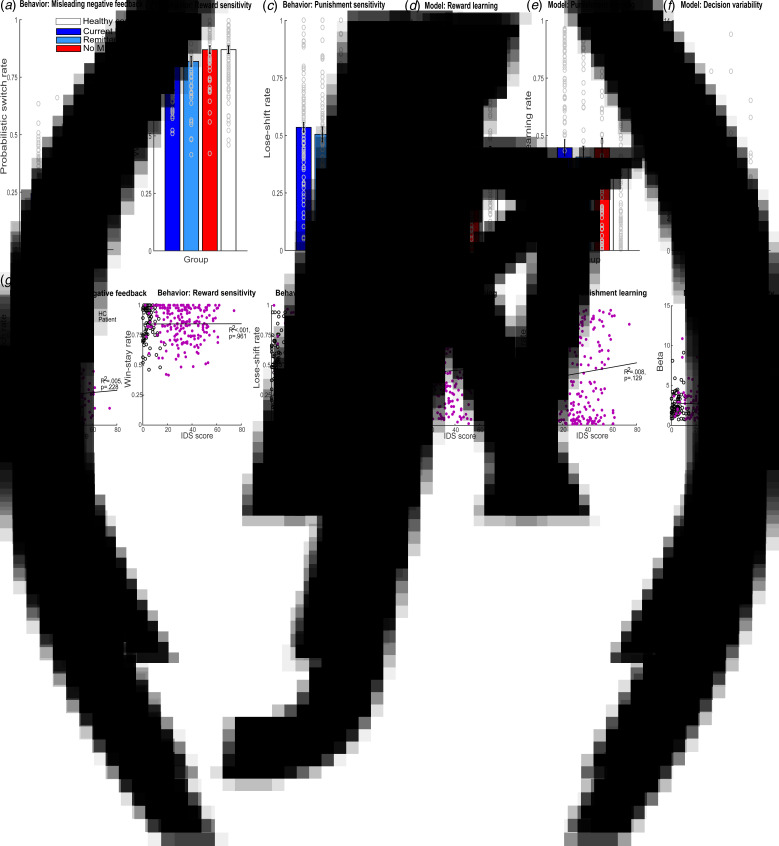
Behavioral and computational results. Pair-wise comparisons of (a) probabilistic switch rate, (b) win-stay rate, (c) lose-shift rate, (d) reward learning rate, (e) punishment learning rate and (f) decision variability with Group as between-subjects factor. Dimensional analyses of (g) probabilistic switch rate, (h) win-stay rate and (i) lose-shift rate, (j) reward learning rate, (k) punishment learning rate and (l) decision variability with IDS score.

Win-stay rate was higher than lose-shift rate [main effect of error type: *F*(1,285) = 46.76, *p* < 0.001, *η*_p_^2^ = 0.141], but did not differ between the groups [main effect of Group: *F*(3,285) = 1.04, *p* = 0.378, *η*_p_^2^ = 0.011], nor was there a significant interaction between Group and error type [*F*(3,285) = 0.64, *p* = 0.589, *η*_p_^2^ = 0.007]. Critically, the groups also did not differ in terms of probabilistic switch rate [*F*(3,285) = 1.73, *p* = 0.161, *η*_p_^2^ = 0.018] (Fig. 3a–c). These results were confirmed by a Bayesian ANOVA, where the Bayes Factor was 12.66 for probabilistic switch rate and 58.82 for error type. This meant that there was strong evidence in favor of the null hypothesis (no difference between the groups) compared with the alternative hypotheses (a difference between the groups).

#### Computational modeling parameters

There was neither difference between reward and punishment learning rate [*F*(1,285) = 0.09, *p* = 0.764, *η*_p_^2^ < 0.001], nor a difference between the four groups [*F*(3,285) = 1.69, *p* = 0.170, *η*_p_^2^ = 0.017] or an interaction [*F*(3,285) = 0.333, *p* = 0.802, *η*_p_^2^ = 0.003] (Figs 3d and 3e). Furthermore, decision variability did not differ significantly between the four groups, *F*(3,285) = 2.16, *p* = 0.093, *η*_p_^2^ = 0.022 (Fig. 3f). These results were confirmed by a Bayesian ANOVA, which showed that the null-hypothesis was 17.24 and 4.5 more likely than the alternative hypothesis for learning rate and decision variability, respectively. This was considered moderate to strong evidence that there was no difference between the groups.

#### Dimensional analyses

In addition to the group-wise comparisons, we investigated the associations of depressive, anxiety, ADHD and autism symptom severity ratings with the outcome measures. There were no significant associations between these ratings and the outcome measures (IDS all *p* > 0.094; ASI all *p* > 0.180; CAARS all *p* > 0.102; AQ all *p* > 0.142) (Fig. 3g–l; online Supplementary Table S3).

### Specificity analyses

#### Probabilistic reversal learning deficits as a function of the other diagnoses

**Fig. 4.**
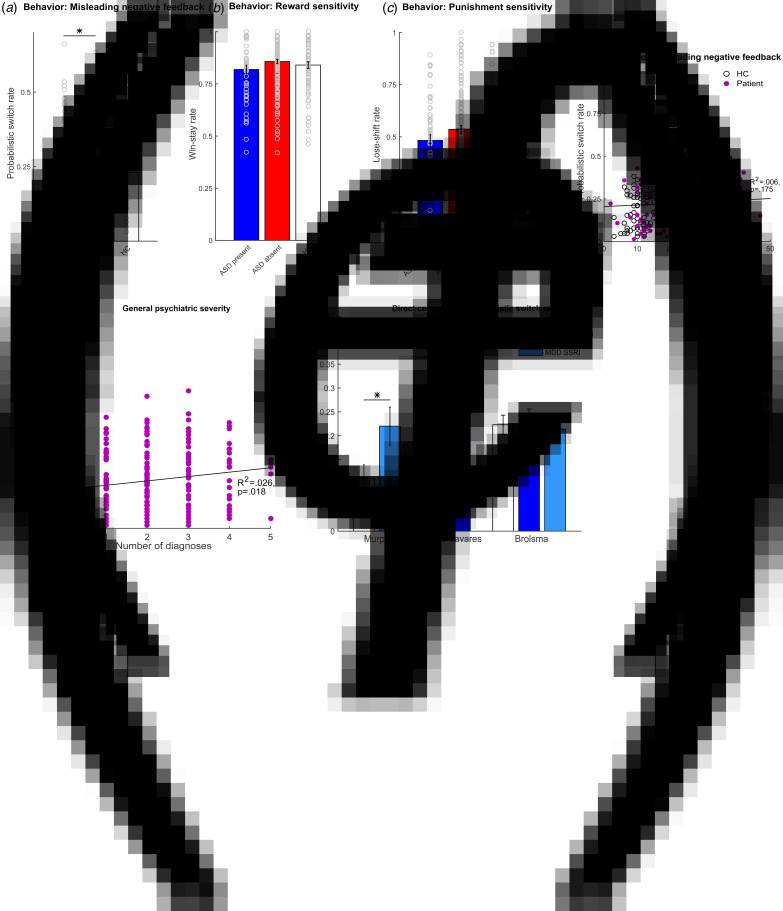
Results from specificity analyses. Pair-wise comparisons between ASD present or absent of (a) probabilistic switch rate, (b) win-stay rate and (c) lose-shift rate. (d) Association of probabilistic switch rate with AQ-50 score, indexing severity of autism symptoms. (e) Association of probabilistic switch rate with general psychiatric severity, as indexed by the number of diagnoses. (f) Direct comparison between mean probabilistic switch rate of Murphy et al. (2003), Taylor Tavares et al. (2008), and the current study.

Next, we repeated the analyses using the other diagnoses (anxiety disorder, addictive disorder, ADHD, ASD) as between-subjects grouping factor (online Supplementary Table S4). Figure 1 shows the overlap between the different disorders. The primary rationale for these analyses was to investigate whether any observed effects of MDD were specific to MDD or extended to other psychiatric disorders. There was no effect of any grouping on the computational modeling parameters. However, there was a significant difference when the group was stratified based on ASD [*F*(2,280) = 3.96, *p* = 0.020, *η*_p_^2^ = 0.028]. Patients with ASD exhibited a higher probabilistic switch rate (i.e. more errors after misleading negative feedback) compared with patients without ASD, *t*(209) = 2.04, *p* = 0.042 [the difference between HC and patients with ASD, and HC and patients without ASD was not significant, *t*(136) = 1.26, *p* = 0.209 and *t*(233) = 0.62, *p* = 0.534 respectively]. However, these effects did not survive correction for multiple comparisons. No other significant effects were found (Figs 4a–d, online Supplementary Table S4).

#### Probabilistic switch rate increases with general psychiatric severity (number of diagnoses)

We did not find effects of medication on our outcome measures of interest (online Supplementary Results). However, there was a significant effect of number of diagnoses on the probabilistic switch rate [*F*(1,215) = 5.67, *p* = 0.018, *η*_p_^2^ = 0.026]; patients with more diagnoses exhibited a higher probabilistic switch rate (Fig. 4e). We found no other effect of general psychiatric severity (online Supplementary Results).

#### Comparison probabilistic switch rate with previous studies

Probabilistic switch rate was not significantly different between HC, MDD patients without SSRI use and MDD patients with SSRI use [main effect of Group: *F*(2,84) = 0.03, *p* = 0.970, *η*_p_^2^ = 0.001]. In Fig. 4f we present the mean probabilistic switch rates of the current study together with those of Murphy et al. (2003) and Taylor Tavares et al. (2008). Additionally, we compared win-stay and lose-shift rate, and reward and punishment learning rate with the results from non-depressed individuals from three other studies (online Supplementary Table S5). On average, our participants perform like those in other studies. Moreover, the degree of individual variability in these measures, as indexed by the standard deviations, also resemble those reported previously.

### Demographic information

There were no significant differences between the four groups in terms of age, gender, IQ, education level, errors on the spatial working memory task, number of perseverative errors, or number of people that reached the learning criterion (Table 1). As expected there was a significant group difference in depressive symptom severity (IDS-SR total score), with lower ratings in HCs than the No MDD [*t*(64.78) = −9.62, *p* < 0.001], Remitted MDD [*t*(66.37) = −16.84, *p* < 0.001] and Current MDD group [*t*(126.94) = −29.07, *p* < 0.001]. Depressive symptom ratings of the No MDD group were lower than of the Remitted MDD group [*t*(103.48) = −2.29, *p* = 0.024] and the Current MDD group [*t*(158) = −8.85, *p* < 0.001], and the Remitted MDD group had lower ratings than the Current MDD group [*t*(154) = −7.1, *p* < 0.001].

**Table 1.** Sample characteristics: Demographic information and group comparisons

	No MDD (*n* = 61)	Remitted MDD (*n* = 55)	Current MDD (*n* = 101)	Healthy controls (*n* = 81)	Group comparisons
Gender	42 m	30 m	62 m	41 m	χ^2^(3) = 5.47,
	19 f	25 f	39 f	40 f	*p* = 0.140
Age, mean (s.d.)	40.6 (14.6)	37.5 (13)	41.5 (14.8)	40.3 (17)	*F*(3,293) = 0.83, *p* = 0.480
IQ, mean (s.d.)	98.2 (12.4)	100.2 (10.8)	98.5 (10.2)	101 (12.4)	*F*(3,291) = 0.99, *p* = 0.397
Education level	None: 6.8%	None: 0%	None: 4%	None: 0%	χ^2^(8) = 15.32, *p* = 0.083
	Low: 15.3%	Low: 11.3%	Low: 16.2%	Low: 7.4%	
	Middle: 44.1%	Middle: 37.7%	Middle: 42.4%	Middle: 40.7%	
	High: 33.9%	High: 50.9%	High: 37.4%	High: 51.9%	
SWM errors, mean (s.d.)	24.5 (20.5)	19.1 (20.5)	26.3 (20.9)	23.5 (28.8)	*F*(3,289) = 1.13, *p* = 0.337
Perseverative errors (s.d.)	9 (6.7)	10 (7.8)	11 (8.2)	10 (7.6)	*F*(3,285) = 0.71, *p* = 0.545
Learning criterion reached	68.9%	65.5%	65.3%	65.4%	χ^2^(3) = 0.26, *p* = 0.968
IDS-SR score, mean (s.d.)	23.0 (14)	28.1 (9.7)	41.3 (11.8)	4.9 (0.4)	*F*(3,290) = 182.4, *p* < 0.001
No of diagnoses^a^				–	χ^2^(10) = 88.33, *p* < 0.001
0	19.7%	–	–		
1	49.2%	14.5%	12.9%		
2	27.9%	49.1%	36.6%		
3	3.3%	29.1%	29.7%		
4	–	7.3%	14.9%		
5	–	–	5.9%		

IDS-SR, Inventory of Depressive Symptomatology – self-report version; SWM, spatial working memory; s.d., standard deviation.

a Total number of diagnoses: the No MDD group included 12 individuals (19.7%) for whom the diagnosis was undefined, but there was a strong suspicion of ADHD and/or ASD at the time of inclusion. These are recorded as having zero diagnoses in the table.

Furthermore, there was a significant difference between the groups in terms of the total number of diagnoses, with fewer diagnoses in the No MDD group than the Remitted MDD group, χ^2^(4) = 38.9, *p* < 0.001, and than the Current MDD group, χ^2^(5) = 63.8, *p* < 0.001. There was no significant difference in the number of diagnoses between the Remitted MDD group and the Current MDD group, χ^2^(4) = 6.3, *p* = 0.176.

## Discussion

In the present study, we assessed the generalizability of the negative learning bias hypothesis of depression from selected depressed patient samples to a large, heterogeneous sample of depressed patients with high levels of specified comorbidities, by measuring learning from punishment *v.* learning from reward. In contrast to previous studies focusing on selected and smaller samples, patients with MDD did not exhibit a negative bias compared with HC in terms of any of the behavioral and computational measures indexing increased learning from punishment (punishment learning rate, lose-shift behavior, probabilistic switch rate). The severity of depressive symptoms was not associated with any of the behavioral or computational model-derived measures.

The negative bias hypothesis is a dominant and enduring account of MDD, which is grounded in evidence from studies using a variety of cognitive tasks, including learning paradigms (Beck, 1986, 2008; Eshel & Roiser, 2010; Gotlib & Joormann, 2010). We find no evidence in support of this negative learning bias account in this naturalistic sample of psychiatric patients. The critical difference with previous studies is the presence of comorbid psychiatric disorders. It is possible that learning deficits that have been associated with the other disorders have an influence during this task as well. For example, increased reward sensitivity in addiction (Nusslock & Alloy, 2017) and atypical reward processing has been found in ADHD (Luman, Oosterlaan, & Sergeant, 2005; Thoma, Edel, Suchan, & Bellebaum, 2015), which has also been associated with motivation deficits in ADHD (Volkow et al., 2011).

Previous studies have generally found decreased reward sensitivity and learning in MDD (Admon & Pizzagalli, 2015), which has been associated with one of the main symptoms of depression, anhedonia (Huys et al., 2013; Pizzagalli, Goetz, Ostacher, Iosifescu, & Perlis, 2008a; Robinson & Chase, 2017). In contrast, we did not find evidence for reduced learning from or insensitivity to reward in patients with depression, nor for an association between the outcome measures and the level of anhedonia symptoms (see online Supplementary Material). However, we did not measure anhedonia with a specific questionnaire and can therefore not draw any definitive conclusions about a possible specific effect of anhedonia (as opposed to a more general effect of depression) on reward learning. It would be interesting to examine this with a questionnaire that measures anhedonia symptoms, such as the Snaith-Hamilton Pleasure Scale (SHAPS, Snaith et al., 1995).

To investigate whether comorbidity and medication status affected our findings, we performed supplementary analyses, selecting a subset of healthy controls and patients that matched the samples of Murphy et al. (2003) and Taylor Tavares et al. (2008) based on age, comorbidity, and medication use (Fig. 4f). Surprisingly, these analyses also revealed no effects on any of the outcome measures indexing negative bias. While comorbidity with anxiety disorders is often not excluded, ADHD or ASD are not mentioned, and possibly not measured. Given the substantial overlap in our sample, it is possible that they are underdiagnosed in other studies. Furthermore, we performed rigorous screenings on these disorders for our healthy control group, which was quite large compared to other studies. Noteworthy, and in line with our results, two recent studies using computational modeling also did not find enhanced punishment learning rate in MDD (Huys et al., 2013; Kunisato et al., 2012).

Additionally, we examined whether the negative learning bias hypothesis is specific to depression, or whether it extends to other psychiatric diagnoses. When the sample was divided based on the other major psychiatric disorders, we found marginally increased switching after misleading negative feedback in patients with ASD, an effect generally consistent with a previous finding of increased attention to negative social-emotional images in ASD (Unruh, Bodfish, & Gotham, 2018). However, we note that caution is warranted when interpreting these findings for two reasons. First, the marginal effects did not survive our significance threshold when correcting for multiple comparisons, which is appropriate particularly given the exploratory nature of these supplementary analyses. Second, the probabilistic switch rate in the ASD group did not differ from those of HC. These findings might suggest the presence of underestimated and unmeasured comorbidity in previous samples.

Interestingly, we also found evidence indicating that patients with more disorders responded more towards misleading negative feedback. However, the number of diagnoses did not have an effect on any of the other measures indexing negative bias.

We had a limited number of exclusion criteria (e.g. current non-affective psychotic disorder or mental retardation). Naturalistic sampling as done in the current study is an advantage, but can also have its drawbacks. There are several factors which are more easily monitored in a smaller sample. For instance, while we did record medication use, we did not specifically examine the effects of daily dosage and the number of years used. Additionally, some patients had experience with one or more behavioral therapies, which might also influence behavior. Future studies should attempt to control these factors, or potentially investigate their effect in heterogeneous clinical samples. Another limitation of the current study is that negative learning bias is measured with only one task. We acknowledge that the present lack of a negative bias might not generalize to other tasks such as those measuring emotional processing (Everaert et al., 2017; Gaddy & Ingram, 2014; LeMoult & Gotlib, 2019; Mathews & MacLeod, 2005). The use of this particular task was motivated by previous literature, enabling us to compare these results with previous studies that were also done in smaller groups of patients with MDD (Murphy et al., 2003; Taylor Tavares et al., 2008). Of note, in a follow-up study in which we employed a slightly different (deterministic) reversal-learning task, we again were unable to provide evidence for changes in punishment (or reward) learning (Brolsma et al., Submitted). Finally, one should note that we used one prior to fit the data of both the patients and the control participants. This is considered a stricter test of the differences, as using the use of two (or more) priors increases the chance of false positives. Yet, this approach may increase the chance of false negatives, and such different procedures should be addressed in future work. Noteworthy, in line with our modeling results, we did not observe any difference in the behavioral outcome measures in our data.

Our results have implications for the negative learning bias hypothesis of MDD. We do not find any evidence for a negative learning bias in MDD. If anything, our results suggest that negative learning bias is associated with ASD. We cannot exclude the possibility that previously reported negative learning bias in patients with MDD reflects comorbid (undiagnosed) ASD, because screening for ASD diagnosis in those previous studies (Admon et al., 2017; Elliott et al., 1997; Liu et al., 2017; Robinson et al., 2012; Taylor Tavares et al., 2008) remained unreported. However, and importantly, the evidence for negative bias in depression in other cognitive domains such as attention, interpretation and memory is rather robust (Everaert et al., 2017; Gaddy & Ingram, 2014; Peckham, McHugh, & Otto, 2010), and has more recently been extended to other psychiatric disorders (e.g. anxiety disorders, substance abuse disorders, ADHD symptoms, ASD) (Bar-Haim, Lamy, Pergamin, Bakermans-Kranenburg, & van IJzendoorn, 2007; Field, Munafò, & Franken, 2009; Gotham, Unruh, & Lord, 2015; Unruh et al., 2018; Vrijsen et al., 2017, 2018). The expression of negative bias may depend on the cognitive domain studied, and remain limited to affective domains, without impacting learning. Understanding the complexity of cognitive mechanisms underlying depression with the purpose of predicting psychiatric vulnerability therefore requires investigating symptomatology in large and naturalistic samples.

## Supplementary material

For supplementary material accompanying this paper visit https://doi.org/10.1017/S0033291720001956. click here to view supplementary material

## References

[ref1] Admon, R, Kaiser, RH, Dillon, DG, Beltzer, M, Goer, F, & Pizzagalli, DA. (2017). Dopaminergic enhancement of striatal response to reward in major depression. American Journal of Psychiatry, 174(4), 378–386. 10.1176/appi.ajp.2016.16010111 PMC5378658 27771973

[ref2] Admon, R., & Pizzagalli, D. A. (2015). Dysfunctional reward processing in depression. Current Opinion in Psychology, 4, 114–118. 26258159 10.1016/j.copsyc.2014.12.011 PMC4525714

[ref3] American Psychiatric Association. (2013). Diagnostic and statistical manual of mental disorders (DSM-5®) American Psychiatric Pub. Cambridge Cognition 2019: CANTAB® [Cognitive assessment software]. Cambridge Cognition (2019). All rights reserved. www.cantab.com.

[ref4] Bar-Haim, Y., Lamy, D., Pergamin, L., Bakermans-Kranenburg, M. J., & van IJzendoorn, M. H. (2007). Threat-related attentional bias in anxious and nonanxious individuals: A meta-analytic study. Psychological Bulletin, 133, 1–24. 17201568 10.1037/0033-2909.133.1.1

[ref5] Baron-Cohen, S., Wheelwright, S., Skinner, R., Martin, J., & Clubley, E. (2001). The autism-Spectrum quotient (AQ): Evidence from Asperger Syndrome/high-functioning autism, males and females, scientists and mathematicians. Journal of Autism and Developmental Disorders, 31, 5–17. 11439754 10.1023/a:1005653411471

[ref6] Beck, A. T. (1986). Cognitive models of depression. Journal of Cognitive Psychotherapy, 1, 5–37.

[ref7] Beck, A. T. (2008). The evolution of the cognitive model of depression and its neurobiological correlates. American Journal of Psychiatry, 165, 969–977. 10.1176/appi.ajp.2008.08050721 18628348

[ref8] Brolsma, S. C. A., Vassena, E., Vrijsen, J. N., Sescousse, G., Collard, R. M., van Eijndhoven, P. F., … Cools, R. Negative learning bias in depression revisited: Enhanced neural response to surprising reward across psychiatric disorders. Manuscript submitted for publication. 10.1016/j.bpsc.2020.08.011 33082119

[ref9] Conners, C., Erhardt, D., & Sparrow, E. (1999). Adult ADHD rating scales: Technical manual. North Tonawanda: MHS.

[ref10] Cools, R., Barker, R. A., Sahakian, B. J., & Robbins, T. W. (2001). Enhanced or impaired cognitive function in Parkinson's disease as a function of dopaminergic medication and task demands. Cerebral Cortex, 11, 1136–1143. 11709484 10.1093/cercor/11.12.1136

[ref11] den Ouden, H. E. M., Daw, N. D., Fernandez, G., Elshout, J. A., Rijpkema, M., Hoogman, M., … Cools, R. (2013). Dissociable effects of dopamine and serotonin on reversal learning. Neuron, 80, 1090–1100. 24267657 10.1016/j.neuron.2013.08.030

[ref12] Elliott, R., Sahakian, B. J., Herrod, J. J., Robbins, T. W., & Paykel, E. S. (1997). Abnormal response to negative feedback in unipolar depression: Evidence for a diagnosis specific impairment. Journal of Neurology, Neurosurgery, and Psychiatry, 63, 74–82. 10.1136/jnnp.63.1.74 PMC2169625 9221971

[ref13] Eshel, N., & Roiser, J. P. (2010). Reward and punishment processing in depression. Biological Psychiatry, 68, 118–124. 20303067 10.1016/j.biopsych.2010.01.027

[ref14] Everaert, J., Podina, I. R., & Koster, E. H. W. (2017). A comprehensive meta-analysis of interpretation biases in depression. Clinical Psychology Review, 58, 33–48. 28974339 10.1016/j.cpr.2017.09.005

[ref15] Field, M., Munafò, M. R., & Franken, I. H. A. (2009). A meta-analytic investigation of the relationship between attentional bias and subjective craving in substance abuse. Psychological Bulletin, 135, 589–607. 19586163 10.1037/a0015843 PMC2999821

[ref16] First MB, Spitzer RL, Gibbon M, & Williams JBW (1996). Structured clinical interview for DSM-IV axis I disorders research version *(*SCID-I*)*. New York, NY: Biometrics Research, New York State Psychiatric Institute.

[ref17] Frank, M. J., Moustafa, A. A., Haughey, H. M., Curran, T., & Hutchison, K. E. (2007). Genetic triple dissociation reveals multiple roles for dopamine in reinforcement learning. Proceedings of the National Academy of Sciences of the United States of America, 104, 16311–6. 17913879 10.1073/pnas.0706111104 PMC2042203

[ref18] Gaddy, M. A., & Ingram, R. E. (2014). A meta-analytic review of mood-congruent implicit memory in depressed mood. Clinical Psychology Review, 34, 402–416. 24980699 10.1016/j.cpr.2014.06.001

[ref19] Goldberg, D., & Fawcett, J. (2012). The importance of anxiety in both major depression and bipolar disorder. Depression and Anxiety, 29, 471–478. 22553107 10.1002/da.21939

[ref20] Gotham, K., Unruh, K., & Lord, C. (2015). Depression and its measurement in verbal adolescents and adults with autism spectrum disorder. Autism: The International Journal of Research and Practice, 19, 491–504. 24916450 10.1177/1362361314536625 PMC4467786

[ref21] Gotlib, I. H., & Joormann, J. (2010). Cognition and depression: Current Status and future directions. Annual Review of Clinical Psychology, 6, 285. 10.1146/annurev.clinpsy.121208.131305 PMC2845726 20192795

[ref22] Harlé, K. M., Guo, D., Zhang, S., Paulus, M. P., & Yu, A. J. (2017). Anhedonia and anxiety underlying depressive symptomatology have distinct effects on reward-based decision-making. PLOS ONE, 12, e0186473. 29059254 10.1371/journal.pone.0186473 PMC5653291

[ref23] Hoekstra, R. A., Bartels, M., Cath, D. C., & Boomsma, D. I. (2008). Factor structure, reliability and criterion validity of the Autism-Spectrum Quotient (AQ): A study in Dutch population and patient groups. Journal of Autism and Developmental Disorders, 38, 1555–1566. 18302013 10.1007/s10803-008-0538-x PMC2516538

[ref24] Huys, Q. J., Pizzagalli, D. A., Bogdan, R., & Dayan, P. (2013). Mapping anhedonia onto reinforcement learning: A behavioural meta-analysis. Biology of Mood & Anxiety Disorders, 3, 12. 23782813 10.1186/2045-5380-3-12 PMC3701611

[ref25] Ikram, U. Z., Snijder, M. B., Fassaert, T. J. L., Schene, A. H., Kunst, A. E., & Stronks, K. (2015). The contribution of perceived ethnic discrimination to the prevalence of depression. European Journal of Public Health, 25, 243–248. 25416918 10.1093/eurpub/cku180

[ref26] Insel, T., Cuthbert, B., Garvey, M., Heinssen, R., Pine, D. S., Quinn, K., … Wang, P. (2010). Research domain criteria (RDoC): Toward a New classification framework for research on mental disorders. American Journal of Psychiatry, 167, 748–751. 10.1176/appi.ajp.2010.09091379 20595427

[ref27] JASP Team (2019). JASP (Version 0.11.0) [Computer software].

[ref28] Kessler, R. C., Berglund, P., Demler, O., Jin, R., Koretz, D., Merikangas, K. R., … Wang, P. S. (2003). The epidemiology of major depressive disorder. JAMA, 289, 3095. 12813115 10.1001/jama.289.23.3095

[ref29] Kooij, J. J. S., & Francken, M. H. (2010). Diagnostic interview for ADHD in adults 2.0 *(*DIVA 2.0*)*. Amsterdam: Pearson Assessment and Information BV.

[ref30] Kunisato, Y., Okamoto, Y., Ueda, K., Onoda, K., Okada, G., Yoshimura, S., … Yamawaki, S. (2012). Effects of depression on reward-based decision making and variability of action in probabilistic learning. Journal of Behavior Therapy and Experimental Psychiatry, 43, 1088–1094. 22721601 10.1016/j.jbtep.2012.05.007

[ref31] Lamers, F., van Oppen, P., Comijs, H. C., Smit, J. H., Spinhoven, P., van Balkom, A. J. L. M., … Penninx, B. W. J. H. (2011). Comorbidity patterns of anxiety and depressive disorders in a large cohort study. The Journal of Clinical Psychiatry, 72, 341–348. 21294994 10.4088/JCP.10m06176blu

[ref32] LeMoult, J., & Gotlib, I. H. (2019). Depression: A cognitive perspective. Clinical Psychology Review, 69, 51–66. 29961601 10.1016/j.cpr.2018.06.008 PMC11884012

[ref33] Liu, W-H, Valton, V, Wang, L-Z, Zhu, Y-H, & Roiser, JP. (2017). Association between habenula dysfunction and motivational symptoms in unmedicated major depressive disorder. Social Cognitive and Affective Neuroscience, 12, 1520–1533. 28575424 10.1093/scan/nsx074 PMC5629818

[ref34] Luman, M., Oosterlaan, J., & Sergeant, J. A. (2005). The impact of reinforcement contingencies on AD/HD: A review and theoretical appraisal. Clinical Psychology Review, 25, 183–213. 15642646 10.1016/j.cpr.2004.11.001

[ref35] Mathews, A., & MacLeod, C. (2005). Cognitive vulnerability to emotional disorders. Annual Review of Clinical Psychology, 1, 167–195. 10.1146/annurev.clinpsy.1.102803.143916 17716086

[ref36] Murphy, F. C., Michael, A., Robbins, T. W., & Sahakian, B. J. (2003). Neuropsychological impairment in patients with major depressive disorder: The effects of feedback on task performance. Psychological medicine, 33, 455–467. 12701666 10.1017/s0033291702007018

[ref37] National Institute of Mental Health (2008). The national institute of mental health strategic plan. Bethesda, MD: National Institute of Mental Health. 2008 (NIH Publication No. 08-6368). Retrieved from http://www.nimh.nih.gov/about/strategic-planning-reports/index.shtml.

[ref38] Nusslock, R., & Alloy, L. B. (2017). Reward processing and mood-related symptoms: An RDoC and translational neuroscience perspective. Journal of Affective Disorders, 216, 3–16. 28237133 10.1016/j.jad.2017.02.001 PMC6661152

[ref39] Pechtel, P., Dutra, S. J., Goetz, E. L., & Pizzagalli, D. A. (2013). Blunted reward responsiveness in remitted depression. Journal of Psychiatric Research, 47, 1864–1869. 24064208 10.1016/j.jpsychires.2013.08.011 PMC3978009

[ref40] Peckham, A. D., McHugh, R. K., & Otto, M. W. (2010). A meta-analysis of the magnitude of biased attention in depression. Depression and Anxiety, 27, 1135–1142. 21049527 10.1002/da.20755

[ref41] Pizzagalli, D. A., Goetz, E., Ostacher, M., Iosifescu, D. V., & Perlis, R. H. (2008a). Euthymic patients with bipolar disorder show decreased reward learning in a probabilistic reward task. Biological Psychiatry, 64, 162–168. 18242583 10.1016/j.biopsych.2007.12.001 PMC2464620

[ref42] Pizzagalli, D. A., Iosifescu, D., Hallett, L. A., Ratner, K. G., & Fava, M. (2008b). Reduced hedonic capacity in major depressive disorder: Evidence from a probabilistic reward task. Journal of Psychiatric Research, 43, 76–87. 18433774 10.1016/j.jpsychires.2008.03.001 PMC2637997

[ref43] Rescorla, R. A., & Wagner, A. R. (1972). A theory of Pavlovian conditioning: Variations in the effectiveness of reinforcement and nonreinforcement. Classical conditioning II: Current research and theory (pp. 64–99). New York: Appleton-Century-Crofts.

[ref44] Robinson, O. J., & Chase, H. W. (2017). Learning and choice in mood disorders: Searching for the computational parameters of Anhedonia. Computational Psychiatry, 1, 208–233. 29400358 10.1162/CPSY_a_00009 PMC5796642

[ref45] Robinson, O. J., Cools, R., Carlisi, C. O., Sahakian, B. J., & Drevets, W. C. (2012). Ventral striatum response during reward and punishment reversal learning in unmedicated major depressive disorder. The American Journal of Psychiatry, 169, 152–159. 22420038 10.1176/appi.ajp.2011.11010137 PMC5648982

[ref46] Rodriguez, B. F., Bruce, S. E., Pagano, M. E., Spencer, M. A., & Keller, M. B. (2004). Factor structure and stability of the Anxiety Sensitivity Index in a longitudinal study of anxiety disorder patients. Behaviour Research and Therapy, 42, 79–91. 14744525 10.1016/s0005-7967(03)00074-3 PMC3272759

[ref47] Roiser, J. P., & Sahakian, B. J. (2013). Hot and cold cognition in depression. CNS Spectrums, 18, 139–149. 23481353 10.1017/S1092852913000072

[ref48] Rommelse, N. N. J., Geurts, H. M., Franke, B., Buitelaar, J. K., & Hartman, C. A. (2011). A review on cognitive and brain endophenotypes that may be common in autism spectrum disorder and attention-deficit/hyperactivity disorder and facilitate the search for pleiotropic genes. Neuroscience & Biobehavioral Reviews, 35, 1363–1396. 21382410 10.1016/j.neubiorev.2011.02.015

[ref49] Rothkirch, M, Tonn, J, Köhler, S, & Sterzer, P. (2017). Neural mechanisms of reinforcement learning in unmedicated patients with major depressive disorder. Brain, 140, 1147–1157. 28334960 10.1093/brain/awx025

[ref50] Rush, A. J., Gullion, C. M., Basco, M. R., Jarrett, R. B., & Trivedi, M. H. (1996). The inventory of depressive symptomatology (IDS): Psychometric properties. Psychological Medicine, 26, 477. 8733206 10.1017/s0033291700035558

[ref51] Safra, L., Chevallier, C., & Palminteri, S. (2019). Depressive symptoms are associated with blunted reward learning in social contexts. PLOS Computational Biology, 15, e1007224. 31356594 10.1371/journal.pcbi.1007224 PMC6699715

[ref52] Schippers, G., Broekman, T., & Buchholz, A. (2011). MATE 2.1. Manual and Protocol.

[ref53] Schmand, B., Lindeboom, J., & Van Harskamp, F. (1992). De nederlandse leestest voor volwassenen. [the Dutch adult reading test.]. Lisse, The Netherlands: Swets & Zeitlinger.

[ref54] Snaith, R. P., Hamilton, M., Morley, S., Humayan, A., Hargreaves, D., & Trigwell, P. (1995). A scale for the assessment of hedonic tone. The Snaith-Hamilton Pleasure Scale. British Journal of Psychiatry, 167, 99–103. 10.1192/bjp.167.1.99 7551619

[ref55] Swainson, R., Rogers, R. D., Sahakian, B. J., Summers, B. A., Polkey, C. E., & Robbins, T. W. (2000). Probabilistic learning and reversal deficits in patients with Parkinson's disease or frontal or temporal lobe lesions: Possible adverse effects of dopaminergic medication. Neuropsychologia, 38, 596–612. 10689037 10.1016/s0028-3932(99)00103-7

[ref56] Taylor Tavares, J. V., Clark, L., Furey, M. L., Williams, G. B., Sahakian, B. J., & Drevets, W. C. (2008). Neural basis of abnormal response to negative feedback in unmedicated mood disorders. NeuroImage, 42, 1118–1126. 18586109 10.1016/j.neuroimage.2008.05.049 PMC2745889

[ref57] Thoma, P., Edel, M.-A., Suchan, B., & Bellebaum, C. (2015). Probabilistic reward learning in adults with Attention Deficit Hyperactivity Disorder – An electrophysiological study. Psychiatry Research, 225, 133–144. 25467706 10.1016/j.psychres.2014.11.006

[ref58] Timmer, M. H. M., Sescousse, G., van der Schaaf, M. E., Esselink, R. A. J., & Cools, R. (2017). Reward learning deficits in Parkinson's disease depend on depression. Psychological Medicine, 47, 2302–2311. 28374660 10.1017/S0033291717000769

[ref59] Unruh, K. E., Bodfish, J. W., & Gotham, K. O. (2018). Adults with autism and adults with depression show similar attentional biases to social-affective images. Journal of Autism and Developmental Disorders, 48, 1–12. 29882107 10.1007/s10803-018-3627-5 PMC6286233

[ref60] Volkow, N. D., Wang, G.-J., Newcorn, J. H., Kollins, S. H., Wigal, T. L., Telang, F., … Swanson, J. M. (2011). Motivation deficit in ADHD is associated with dysfunction of the dopamine reward pathway. Molecular Psychiatry, 16, 1147–1154. 20856250 10.1038/mp.2010.97 PMC3010326

[ref61] Vrijsen, J. N., Tendolkar, I., Onnink, M., Hoogman, M., Schene, A. H., Fernández, G., … Franke, B. (2018). ADHD symptoms in healthy adults are associated with stressful life events and negative memory bias. ADHD Attention Deficit and Hyperactivity Disorders, 10, 151–160. 29081022 10.1007/s12402-017-0241-x PMC5973996

[ref62] Vrijsen, J. N., van Amen, C. T., Koekkoek, B., van Oostrom, I., Schene, A. H., & Tendolkar, I. (2017). Childhood trauma and negative memory bias as shared risk factors for psychopathology and comorbidity in a naturalistic psychiatric patient sample. Brain and Behavior, 7, e00693. 28638703 10.1002/brb3.693 PMC5474701

[ref63] Vuijk, R. (2016). Nederlands Interview ten behoeve van Diagnostiek Autismespectrumstoornis bij volwassenen *(*NIDA*)*. Amsterdam: Boom Uitgevers.

[ref64] Whitton, A. E., Kakani, P., Foti, D., Van't Veer, A., Haile, A., Crowley, D. J., & Pizzagalli, D. A. (2016). Blunted neural responses to reward in remitted major depression: A high-density event-related potential study. Biological Psychiatry. Cognitive Neuroscience and Neuroimaging, 1, 87–95. 26858994 10.1016/j.bpsc.2015.09.007 PMC4743048

[ref65] World Health Organization. (2017). Depression and Other Common Mental Disorders-Global Health Estimates Retrieved from https://www.who.int/mental_health/management/depression/prevalence_global_health_estimates/en/

