# Supplementary material for: Challenging the negative learning bias hypothesis of depression: reversal learning in a naturalistic psychiatric sample
Source: Psychol Med. 2020 Jun 15;52(2):303–13. doi: 10.1017/S0033291720001956 (PMC8842187; doi:10.1017/S0033291720001956)
Supplement: Supplementary file 1 [file S0033291720001956sup001.docx]

Supplementary information

Challenging the negative learning bias hypothesis of depression: reversal learning in a naturalistic psychiatric sample

Brolsma et al.

Contents

[Methods 2](#_Toc38977682)

[Patient inclusion 2](#_Toc38977683)

[Healthy control participants 3](#_Toc38977684)

[Instructions for the probabilistic reversal learning task 3](#_Toc38977685)

[Computational Model 4](#_Toc38977686)

[Effect of anhedonia 5](#_Toc38977687)

[Results 5](#_Toc38977688)

[Dimensional analyses 5](#_Toc38977689)

[Stratification based on other diagnoses 6](#_Toc38977690)

[Model fit 6](#_Toc38977691)

[Medication 6](#_Toc38977692)

[Number of diagnoses 6](#_Toc38977693)

[Comparison MDD group without comorbidity 7](#_Toc38977694)

[Specific comparison of the negative learning bias measures 7](#_Toc38977695)

[Results in participants who passed the learning criterion 8](#_Toc38977696)

[Anhedonia symptoms do not influence behavior on the probabilistic reversal learning task 8](#_Toc38977697)

[Figure S1 9](#_Toc38977698)

[Tables 10](#_Toc38977699)

[References 16](#_Toc38977700)

# Methods

## Patient inclusion

All outpatients completed the online questionnaires before the first visit as part of the standard clinical diagnostic procedure (Table S1). During the first appointment, DSM-IV classifications of depressive disorders (major depressive disorder (MDD) and dysthymia), anxiety disorders, addictive disorders and attention-deficit/hyperactivity disorder (ADHD) were assessed. DSM-5 criteria were used for autism spectrum disorders (ASD). MDD and anxiety disorders were assessed by sections A, B, C, D and F of the Structured Clinical interview for DSM-IV AXIS I Disorder (SCID-I; First et al. 1996). ADHD and ASD were assessed with a two-step diagnostic procedure. First screening questionnaires were used to detect high-risk patients. If there was a positive screening for ADHD (ASRS-short form; cut-off > 3.6 items) and/or ASD (AQ-50; cut-off > 25/50 items), the Diagnostic Interview for Adult ADHD, second edition (DIVA 2.0; Kooij & Francken 2010) and/or the Dutch Diagnostic Interview for Adult Autism Spectrum Disorders (NIDA; Vuijk 2016) were administered at a later appointment as part of the standard clinical procedure. Both the DIVA and NIDA were completed in the presence of a partner and/or family member of the patient to be able to retrospectively and collaterally ascertain information on a broad range of symptoms in childhood and adulthood. This procedure led to inclusion of some participants with a suspicion of ADHD or ASD at time of inclusion in the study, but for which a diagnosis could not be established due to patients not finishing the diagnostic process at this point. When we compared the groups, we included these participants in the No MDD group. Finally, for the addictive disorders we used the Measurements in the Addictions for Triage and Evaluation and criminality (MATE-Crimi; Schippers *et al*. 2011; subsections 1, 3, 4, 9 and Q1). All interviews were done by well-trained clinicians.

In Table S2 we present the total number of MIND-Set patients during the inclusion period, and the number of participants that were included in the current study.

## Healthy control participants

Healthy control participants were approached via databases of the department’s previous studies, advertisement in newspapers, social media, websites, and via the Radboud Research Participation System of the Radboud University Faculty of Social Sciences, as well as verbally through the researchers’ own network.

All healthy participants underwent a telephonic screening where the SCID-I and MATE-Crimi criteria for lifetime MDD, anxiety disorders and addictive disorders were assessed. The ASRS and AQ-50 were used as screening measures for ADHD and ASD respectively. To be included as healthy control, participants could not score higher than 3 out of 6 on the ASRS, or higher than 26 out of 50 on the AQ-50, which are established cut-offs (Baron-Cohen *et al.* 2001; Ustun *et al.* 2017).

Other exclusion criteria for control participants were the use of more than 14 units of alcohol per week, regular drug use (once a week or more, except for nicotine) or benzodiazepine use, existence of a neurological disorder of the central nervous system or current malignancy.

## Instructions for the probabilistic reversal learning task

Participants received a verbal instruction and were shown the following instructions on the computer screen in Dutch before starting the task:

*“On the screen two colored patterns are presented: one yellow, one blue. One of these colors is correct more often than the other one and the computer will tell you whether your choice was correct or incorrect. Choose the color which tends to be correct more often. You have to find out by trial and error which color that is. On certain moments the rule can change, i.e. the other color is now correct more often. Then, switch your response to that color. This can happen one or more times during the task”*

## Computational Model

We tested negative learning bias, using the established Reward-Punishment (RP) model (Frank *et al.* 2007) (equation 1).

$V_{s,t}=\left\{\begin{matrix} \begin{matrix} V_{s,t-1}+\alpha^{rew}(\lambda_{t-1}-V_{s,t-1}) \\ V_{s,t-1}+\alpha^{pun}(\lambda_{t-1}-V_{s,t-1}) \end{matrix} & \begin{matrix} if \lambda_{t-1}=1\text{ } \\ if \lambda_{t-1}=-1 \end{matrix} \end{matrix}\right.$ (Equation 1)

$$V_{\sim s,t}=V_{\sim s,t-1}$$

In this model, the expected value of chosen stimulus *S* at trial $t$($V_{s,t}$) is updated based on the prediction error, i.e. the difference between the outcome of the previous trial ($\lambda_{t-1}\in\{-1,1\}$, either -1 for punishment, or 1 for reward, and the expected value of the previous trial (V_s,t-1_). The learning rate α modulates how quickly the expected value is updated by changing the weight on the prediction error. For the reward trials, the expected value is updated using reward learning rate $\alpha^{rew}$and for punishment trials it is updated using punishment learning rate $\alpha^{pun}$. The value of the unchosen stimulus remains unchanged.

The probability of a choosing each of the two stimuli on their expected values was computed with a softmax function (equation 2): for a set of given stimulus values Y and B, for yellow and blue squares, the probability of choosing option Y in the next trial is as follows:

$p(s_{t+1}=Y)=\frac{\exp(\beta V_{s=Y,t+1})}{\sum_{j} \exp(\beta V_{s=j,t+1})}$ (Equation 2)

Where $p(s_{t+1}=B)=1-P(s_{t+1}=Y)$ and $\beta$ affected choice strategies.

The free parameters ($\theta=[\alpha^{rew},\alpha^{pun},\beta]$) were fitted to the choices of each individual participant using a hierarchical fitting procedure with two consecutive steps (Piray *et al.* 2019). In this estimation, we used initial Gaussian priors. Then, according to the theoretical constraints of the parameters, $\alpha^{rew},\alpha^{pun}\in[0,1]$ and $\beta\in[0,+\infty)$, sigmoid and exponential transformations were applied to the learning rates and beta respectively. We tested recoverability of the parameters using the following procedure: First, we simulated 100 datasets with each participant’s estimated parameters. We next fitted the model to each of these simulated datasets. The recovered parameters were then averaged within each subject (100 stimulations), and correlated with the real data.

## Effect of anhedonia

Previous studies have demonstrated that the consistency with which participants choose an action, given the value that was assigned to that action (i.e. decision variability) was associated positively with anhedonia symptom severity (Huys *et al.* 2013; Robinson & Chase 2017). Here we asked whether performance on the PRL task might also be associated with anhedonia. Although there was no specific questionnaire included in our study to measure anhedonia symptoms, we approximated this with a set of items from the Outcome Questionnaire (OQ-45, Lambert *et al.* 1996) which was previously established, based on a factor analysis, to contribute to an anhedonia factor (Minami *et al.* 2009; items: 3, 13, 20, 21, 24, 31, 43; example questions: “I am not interested in anything”, “I am satisfied with my life”). The associations between the total score of these items and the various outcome measures were assessed with Spearman correlations.

# Results

## Dimensional analyses

When controlling for age, gender and SWM errors, there were no significant Spearman partial correlations between the questionnaire scores and the outcome measures (Table S3).

## Stratification based on other diagnoses

Table S4 shows the results when the patient sample was divided based on additional diagnoses next to MDD. The different behavioral and computational outcome measures were analyzed with separate ANOVAs for each diagnosis, with age, gender and working memory capacity as covariates. There was no significant effect of anxiety (present, absent, HC), addiction (present, absent, HC) or ADHD (present, absent, HC) on any of the outcome measures (Table S4).

## Model fit

The correlation between estimated and recovered parameters was high: Reward learning rate, r=0.97, *p*<.001; punishment learning rate, r=0.98, *p*<.001, decision variability, r=0.97, *p*<.001.

## Medication

We explored the effects of selective serotonin reuptake inhibitor (SSRI) medication use within the patient sample, by comparing patients with SSRI use (n=54), patients with other medication (n=98), and patients without medication use (n=65). There were no significant effects of Medication use on probabilistic switch rate (*F*(2,214)=.65, *p*=.526), error type (two levels: win-stay and lose-shift, *F*(2,214)=.12, *p*=.889), learning rate (two levels: reward and punishment, (*F*(2,214)=1.42, *p*=.243) or decision variability (*F*(2,214)=.46, *p*=.632).

## Number of diagnoses

Next, we assessed the effects of general psychiatric severity, by examining whether any of the outcome measures varied with the total number of diagnoses. There was a significant effect of general psychiatric severity on probabilistic switch rate (*F*(1, 215)=5.67, *p*=.018, η_p_^2^=.026), where patients with more diagnoses had a higher probabilistic switch rate. There was no significant effect of number of diagnoses on error type (two levels: win-stay and lose-shift, *F*(1,215)=.13, *p*=.723), learning rate (two levels: reward and punishment, *F*(1,215)=.62, *p*=.433) or decision variability (*F*(1,215)=.89, *p*=.346).

## Comparison MDD group without comorbidity

There was no significant difference between healthy controls (n=63), clean MDD patients without SSRI use (n=14) and clean MDD patients with SSRI use (n=10) on error type (two levels: win-stay and lose-shift, *F*(2,84)=1.29, *p*=.282), probabilistic switch rate (*F*(2,84)=.03, *p*=.970), learning rate (two levels: reward and punishment, *F*(2,84)=.82, *p*=.444) or decision variability (*F*(2,84)=1.86, *p*=.162).

## Specific comparison of the negative learning bias measures

Direct comparisons also revealed no difference between the Current MDD and HC groups on probabilistic switch rate (*t*(180)=0.62, *p*=.537), lose-shift rate (*t*(180)=0.38, *p*=.705), or punishment learning rate (*t*(180)=1.65, *p*=.101). There was a marginal, subthreshold difference between probabilistic switch rate in the Current MDD and that in No MDD group, but this did not reach significance (*t*(160)=1.96, *p*=.052), particularly not after correction for multiple testing. The apparent trend for the group difference on probabilistic switch rate did not reflect a difference between HC and MDD, thus not weakening the main conclusion. Instead it was driven by a lower probabilistic switch rate in the No MDD compared with Current MDD, although this was not robustly seen in any other analysis, which warrants caution in interpretation. There was no difference between the Current MDD and No MDD groups on lose-shift rate (*t*(160)=0.64, *p*=.525) or punishment learning rate (*t*(160)=0.03, *p*=.978). There was also no difference between the Current MDD and Remitted MDD groups on probabilistic switch rate (*t*(154)=0.93, *p*=.804), lose-shift rate (*t*(154)=0.80, *p*=.423) or punishment learning rate (*t*(154)=0.69, *p*=.490).

## Results in participants who passed the learning criterion

We assessed whether there were any group differences when only the participants who reached the learning criterion were selected (8 consecutive correct responses; No MDD n= 42, Remitted MDD n=34, Current MDD n=64, HC n=52). There were no Group differences on probabilistic switch rate (*F*(3, 185)=2.23, *p*=.086), error type (two levels: win-stay and lose-shift, *F*(3,185)=0.12, *p*=.949), learning rate (two levels: reward and punishment, *F*(3,185)=0.45, *p*=.716) or decision variability (*F*(3,185)=1.60, *p*=.191). Furthermore, there were no significant associations between the level of depressive symptoms (i.e. the IDS total score) and the outcome measures (all *p*>.335, r_s_<.07).

## Anhedonia symptoms do not influence behavior on the probabilistic reversal learning task

There were no significant associations between anhedonia symptoms (as measured with 7 items from the OQ-45) and probabilistic switch rate (*p*=.421), win-stay-rate (*p*=.936), lose-shift rate (*p*=.601), reward learning rate (*p*=.216), punishment learning rate (*p*=.171), or decision variability (*p*=.174).

# Figure S1


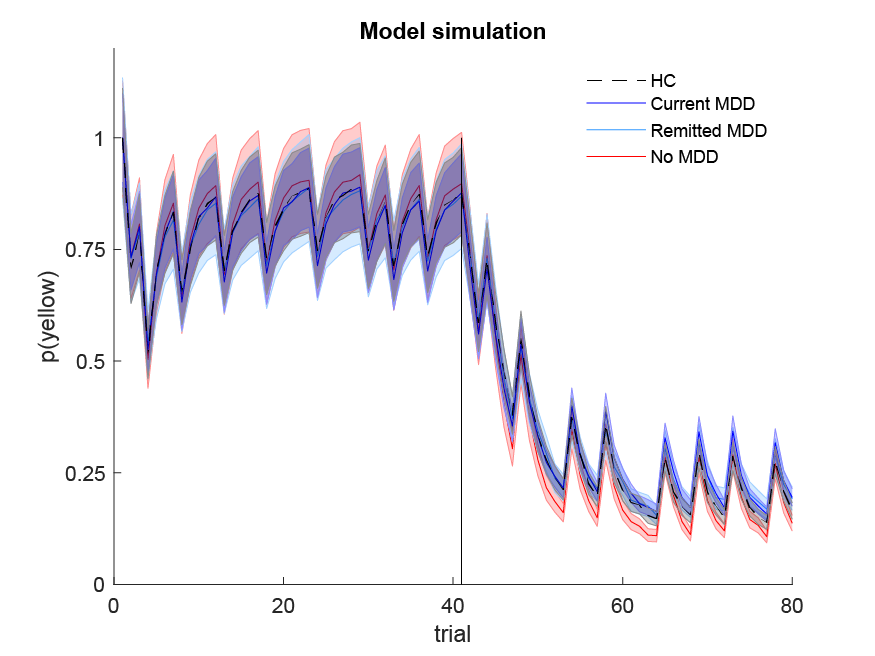


**Figure S1. Simulated trial-by-trial data per group.** The simulated trial-by-trial probability of choosing yellow (the square that was chosen on the first trial) per group. Shade represents the SEM. At trial 41 the contingencies were reversed.

# Tables

**Table S1. Overview of different phases and measures in MIND-Set study.**

| At-home questionnaires (standard clinical procedure) | **Adult ADHD Self-Report Scale*** (ASRS; Ustun *et al*. 2017), **Anxiety Sensitivity Index** (ASI; Rodriguez *et al.* 2004), **Autism-spectrum Quotient** (AQ-50; Baron-Cohen *et al*. 2001; Hoekstra *et al*. 2008), **Conners’ Adult ADHD Rating Scale** (CAARS; Conners *et al.* 1999), Family Interview for Genetic Studies (National Institute of Mental Health 1992), **Inventory of Depressive Symptomatology self-report version** (IDS-SR; Rush *et al.* 1996), MOS Short-Form General Health Questionnaire 20 (Ware & Sherbourne 1992), Outcome Questionnaire-45 (Lambert *et al.* 1996), Personality Inventory for DSM-5 - brief form (Krueger *et al.* 2012), **socio-demographic information** about age, gender and level of education (Stronks *et al.* 2013), and WHO-Disability Assessment Schedule 2.0 (Chwastiak & Von Korff 2003). |
| --- | --- |
| Diagnostic procedure  (standard clinical procedure) | **Sections A, B, C, D and F of the Structured Clinical Interview for DSM-IV Axis I Disorders** (First *et al.* 1996)  **Subsections 1, 3, 4, 9 and Q1 of the Measurements in the Addictions for Triage and Evaluation and criminality** (Schippers *et al.* 2011),  Presence of somatic diseases (Kriegsman *et al.* 1996)  Blood samples: assays included albumin, creatinine, gene expression (DNA, RNA, epigenetics), glucose level, HDL & LDL cholesterol, hemoglobin, hematocrit, inflammatory markers, kidney function, liver function, thyroid function and triglycerides. |

| Neuropsychological assessment (at the Department of Psychiatry, Radboudumc) | Tasks: self-referent encoding task (Hammen & Zupan 1984), the alertness, go no-go and incompatibility tasks of the normed Tests for Attentional Performance Version 2.3 (TAP 2.3 (Zimmermann & Fimm 2002), **Dutch reading test** (National Adult Reading Test score (NART), Dutch version, Schmand *et al.* 1992), **the probabilistic reversal learning task** (den Ouden *et al.* 2013) the motor-screening, **spatial working memory**, and intra-extra dimensional set shift tasks from the Cambridge Neuropsychological Test Automated Battery (CANTAB; Sahakian & Owen 1992), an attentional bias and emotion recognition task (Isaac *et al.* 2014) using an eye-tracker system  Questionnaires: one question about the relation between self-image and the disorder, a food intake questionnaire, Behavior Rating Inventory of Executive Functioning –Adult version (Hocking *et al.* 2015), NEMESIS-childhood trauma questionnaire (de Graaf *et al.* 2010), Perseverative Thinking Questionnaire (Ehring *et al.* 2011) and Toronto Alexithymia Scale-20 (Bagby *et al.* 1994),  Biological measures: Hair sample for cortisol, stool sample for microbiome |
| --- | --- |
| Magnetic resonance imaging (MRI) session | Anatomical scan; functional MRI scans |

* Measures in bold were used in the current study.

**Table S2. Overview diagnoses MIND-Set patients.** The numbers show that there was no bias towards one disorder in the patients who did not want to participant in the study.

|  | Total sample (%)  N=311 | No neuropsychological assessment (%)  N=94 | Current sample (%)  N=217 |
| --- | --- | --- | --- |
| Current depression | 133 (43) | 32 (34) | 101 (46) |
| Remitted depression | 165 (53) | 43 (46) | 122 (56) |
| Anxiety disorder | 95 (31) | 28 (30) | 67 (31) |
| Addictive disorder | 61 (20) | 22 (24) | 39 (18) |
| ADHD | 96 (31) | 19 (20) | 77 (35) |
| ASD | 65 (21) | 8 (9) | 57 (26) |
| No current disorder* | 16 (5) | 5 (5) | 12 (6) |

*We included 16 patients in the total sample for which the diagnosis was undefined, but there was strong suspicion of ADHD or ASD at the time of inclusion, and were therefore reported as having no current disorder. Twelve of them participated in the neuropsychological assessment.

**Table S3. Significance levels from partial Spearman correlations between the questionnaire scores and the outcome measures.**

|  | **Prob switch rate** | **Win-stay rate** | **Lose-shift rate** | **Reward learning rate** | **Punishment learning rate** | **Decision variability** |
| --- | --- | --- | --- | --- | --- | --- |
| **IDS-SR** | *p*=.489 | *p*=.793 | *p*=.741 | *p*=.129 | *p*=.094 | *p*=.186 |
| **ASI** | *p*=.870 | *p*=.299 | *p*=.754 | *p*=.242 | *p*=.180 | *p*=.729 |
| **CAARS** | *p*=.561 | *p*=.883 | *p*=.754 | *p*=.163 | *p*=.102 | *p*=.275 |
| **AQ-50** | *p*=.142 | *p*=.849 | *p*=.257 | *p*=.696 | *p*=.636 | *p*=.658 |

**Table S4. Patient sample based on other diagnoses absent or present.**

|  | **Anxiety** | **Addiction** | **ADHD** | **ASD** |
| --- | --- | --- | --- | --- |
| **Probabilistic switch rate** | *F*(2,286)=.47, *p*=.623 | *F*(2,277)=.40, *p*=.670 | *F*(2,280)=2.89, *p*=.057 | *F*(2,280)=3.96, *p*=.020* |
| **Error type** | *F*(2,286)=.82, *p*=.443 | *F*(2,277)=.10, *p*=.909 | *F*(2,280)=.31, *p*=.736 | *F*(2,280)=.123, *p*=.885 |
| **Learning rate** | *F*(2,286)=.36, *p*=.695 | *F*(2,277)=.58, *p*=.563 | *F*(2,280)=.42, *p*=.658 | *F*(2,280)=.50, *p*=.610 |
| **Decision variability** | *F*(2,286)=1.28, *p*=.281 | *F*(2,277)=.39, *p*=.681 | *F*(2,280)=.54, *p*=.582 | *F*(2,280)=.44, *p*=.642 |

The * indicates uncorrected p<0.05.

**Table S5. Comparison values with other studies.**

|  | Win-stay  mean, SD | Lose-shift  mean, SD | Reward learning rate  mean, SD | Punishment learning rate  mean, SD |
| --- | --- | --- | --- | --- |
| Brolsma (Current study) | HC: 0.84, 0.14  MDD: 0.85, 0.13 | HC: 0.52, 0.21  MDD: 0.53, 0.22 | HC: 0.43, 0.27  MDD: 0.50, 0.26 | HC: 0.36, 0.33  MDD: 0.45, 0.35 |
| Dombrovski *et al.* 2010 | - | - | HC: 0.42, 0.37 | HC: ~0.16 |
| den Ouden *et al.* 2013 | HC: 0.81, 0.16 | HC: 0.57, 0.22 | - | - |
| Culbreth *et al.* 2016 | HC: 0.8, 02 | HC: 0.4, 0.1 | - | - |

~ Mean and SD not available, mean estimated from bar plots.

References

**Bagby RM, Parker JDA, Taylor GJ** (1994). The twenty-item Toronto Alexithymia scale—I. Item selection and cross-validation of the factor structure. *Journal of Psychosomatic Research* **38**, 23–32.

**Baron-Cohen S, Wheelwright S, Skinner R, Martin J, Clubley E** (2001). The Autism-Spectrum Quotient (AQ): Evidence from Asperger Syndrome/High-Functioning Autism, Males and Females, Scientists and Mathematicians. *Journal of Autism and Developmental Disorders* **31**, 5–17.

**Chwastiak LA, Von Korff M** (2003). Disability in depression and back pain: Evaluation of the World Health Organization Disability Assessment Schedule (WHO DAS II) in a primary care setting. *Journal of Clinical Epidemiology* **56**, 507–514.

**Conners C, Erhardt D, Sparrow E** (1999). *Adult ADHD rating scales: Technical manual.*

**Culbreth AJ, Gold JM, Cools R, Barch DM** (2016). Impaired Activation in Cognitive Control Regions Predicts Reversal Learning in Schizophrenia. *Schizophrenia bulletin* **42**, 484–93.

**Dombrovski AY, Clark L, Siegle GJ, Butters MA, Ichikawa N, Sahakian BJ, Szanto K** (2010). Reward/Punishment reversal learning in older suicide attempters. *The American journal of psychiatry* **167**, 699–707.

**Ehring T, Zetsche U, Weidacker K, Wahl K, Schönfeld S, Ehlers A** (2011). The Perseverative Thinking Questionnaire (PTQ): Validation of a content-independent measure of repetitive negative thinking. *Journal of Behavior Therapy and Experimental Psychiatry* **42**, 225–232.

**First MB, Spitzer RL, Gibbon M, Williams JBW** (1996). Structured clinical interview for DSM-IV axis I disorders research version (SCID-I).

**Frank MJ, Moustafa AA, Haughey HM, Curran T, Hutchison KE** (2007). Genetic triple dissociation reveals multiple roles for dopamine in reinforcement learning. *Proceedings of the National Academy of Sciences of the United States of America* **104**, 16311–6.

**de Graaf R, ten Have M, van Dorsselaer S** (2010). *De psychische gezondheid van de Nederlandse bevolking NEMESIS-2: Opzet en eerste resultaten*

**Hammen C, Zupan BA** (1984). Self-schemas, depression, and the processing of personal information in children. *Journal of Experimental Child Psychology* **37**, 598–608.

**Hocking DR, Reeve J, Porter MA** (2015). Characterising the Profile of Everyday Executive Functioning and Relation to IQ in Adults with Williams Syndrome: Is the BRIEF Adult Version a Valid Rating Scale? *PLOS ONE* **10**, e0137628.

**Hoekstra RA, Bartels M, Cath DC, Boomsma DI** (2008). Factor structure, reliability and criterion validity of the Autism-Spectrum Quotient (AQ): a study in Dutch population and patient groups. *Journal of autism and developmental disorders* **38**, 1555–66.

**Huys QJ, Pizzagalli DA, Bogdan R, Dayan P** (2013). Mapping anhedonia onto reinforcement learning: a behavioural meta-analysis. *Biology of mood & anxiety disorders* **3**, 12.

**Isaac L, Vrijsen JN, Rinck M, Speckens A, Becker ES** (2014). Shorter gaze duration for happy faces in current but not remitted depression: Evidence from eye movements. *Psychiatry Research* **218**, 79–86.

**Kooij JJS, Francken MH** (2010). Diagnostic interview for ADHD in adults 2.0 (DIVA 2.0).

**Kriegsman DMW, Penninx BWJH, Van Eijk JTM, Boeke AJP, Deeg DJH** (1996). Self-reports and general practitioner information on the presence of chronic diseases in community dwelling elderly: A study on the accuracy of patients’ self-reports and on determinants of inaccuracy. *Journal of Clinical Epidemiology* **49**, 1407–1417.

**Krueger RF, Derringer J, Markon KE, Watson D, Skodol AE** (2012). Initial construction of a maladaptive personality trait model and inventory for DSM-5. *Psychological Medicine* **42**, 1879–1890.

**Lambert MJ, Burlingame GM, Umphress V, Hansen NB, Vermeersch DA, Clouse GC, Yanchar SC** (1996). The Reliability and Validity of the Outcome Questionnaire. *Clinical Psychology & Psychotherapy* **3**, 249–258.

**Minami T, Davies DR, Tierney SC, Bettmann JE, McAward SM, Averill LA, ... Wampold BE** (2009). Preliminary Evidence on the Effectiveness of Psychological Treatments Delivered at a University Counseling Center. *Journal of Counseling Psychology* **56**, 309–320.

**National Institute of Mental Health** (1992). *Family Interview for Genetic Studies.*

**den Ouden HEM, Daw ND, Fernandez G, Elshout JA, Rijpkema M, Hoogman M, … Cools R** (2013). Dissociable Effects of Dopamine and Serotonin on Reversal Learning. *Neuron* **80**, 1090–1100.

**Piray P, Ly V, Roelofs K, Cools R, Toni I** (2019). Emotionally Aversive Cues Suppress Neural Systems Underlying Optimal Learning in Socially Anxious Individuals. *Journal of neuroscience* **39**, 1445–1456.

**Robinson OJ, Chase HW** (2017). Learning and Choice in Mood Disorders: Searching for the Computational Parameters of Anhedonia. *Computational psychiatry* **1**, 208–233.

**Rodriguez BF, Bruce SE, Pagano ME, Spencer MA, Keller MB** (2004). Factor structure and stability of the Anxiety Sensitivity Index in a longitudinal study of anxiety disorder patients. *Behaviour Research and Therapy* **42**, 79–91.

**Rush AJ, Gullion CM, Basco MR, Jarrett RB, Trivedi MH** (1996). The Inventory of Depressive Symptomatology (IDS): psychometric properties. *Psychological Medicine* **26**, 477.

**Sahakian BJ, Owen AM** (1992). Computerized assessment in neuropsychiatry using CANTAB: discussion paper. *Journal of the Royal Society of Medicine* **85**, 399–402.

**Schippers G, Broekman T, Buchholz A** (2011). MATE 2.1. Manual and protocol.

**Schmand B, Lindeboom J, Van Harskamp F** (1992). De nederlandse leestest voor volwassenen. [the dutch adult reading test.]

**Stronks K, Snijder MB, Peters RJ, Prins M, Schene AH, Zwinderman AH** (2013). Unravelling the impact of ethnicity on health in Europe: the HELIUS study. *BMC Public Health* **13**, 402.

**Ustun B, Adler LA, Rudin C, Faraone S V., Spencer TJ, Berglund P, ... Kessler RC** (2017). The World Health Organization Adult Attention-Deficit/Hyperactivity Disorder Self-Report Screening Scale for DSM-5. *JAMA Psychiatry* **74**, 520.

**Vuijk R** (2016). *Nederlands Interview ten behoeve van Diagnostiek Autismespectrumstoornis bij volwassenen (NIDA)*

**Ware JE, Sherbourne CD** (1992). The MOS 36-Item Short-Form Health Survey (SF-36): I. Conceptual Framework and Item Selection. *Medical Care* **30**, 473–483.

**Zimmermann P, Fimm B** (2002). A test battery for attentional performance. In *Applied Neuropsychology of Attention Theory, Diagnosis and Rehabilitation*, pp124–165. Psychology Press.
